# Photogrammetry Versus Intraoral Scanning in Complete‐Arch Digital Implant Impression: A Systematic Review and Meta‐Analysis

**DOI:** 10.1111/cid.70059

**Published:** 2025-06-07

**Authors:** Alessandro Pozzi, Lorenzo Arcuri, Paolo Carosi, Andrea Laureti, Jimmy Londono, Hom‐Lay Wang

**Affiliations:** ^1^ Department of Clinical Science and Translational Medicine University of Rome Tor Vergata Rome Italy; ^2^ Department of Periodontics & Oral Medicine University of Michigan School of Dentistry Ann Arbor Michigan USA; ^3^ Department of Restorative Sciences Augusta University Augusta Georgia USA; ^4^ Department of Restorative Dentistry and Biomaterials Sciences Harvard School of Dental Medicine Boston Massachusetts USA; ^5^ Department of Life Science, Health and Health Professions Link Campus University Rome Italy; ^6^ Department of Chemical Science and Technologies University of Rome Tor Vergata Rome Italy; ^7^ University Clinics for Dental Medicine University of Geneva Geneva Switzerland; ^8^ Department of Restorative Sciences Dental College of Georgia Augusta Georgia USA

**Keywords:** dental implant, digital impression, full arch, intraoral scanner, IOS, SPG, stereophotogrammetry

## Abstract

**Statement of the Problem:**

The application of digital impressions for complete‐arch implant supported fixed dental prostheses (FDP) remains controversial, and data from a systematic review with meta‐analysis comparing intraoral scanning (IOS) and stereophotogrammetry (SPG) remain limited.

**Purpose:**

To evaluate and compare the accuracy of currently available digital technologies, specifically IOS and SPG, in capturing complete‐arch implant impressions.

**Materials and Methods:**

An electronic and manual search was conducted on May 4, 2024, across PubMed, Embase, and Cochrane CENTRAL databases following PRISMA guidelines. The search targeted studies (excluding case reports) that assessed the in vivo, in vitro, or ex vivo accuracy of IOS and SPG for complete‐arch implant impressions. Two investigators screened eligible studies using the QUADAS‐2 tool. Accuracy was the primary outcome, including linear, angular, surface deviations, and inter‐implant distance. Three meta‐analyses were performed on angular deviations, trueness, and surface deviations, trueness, and precision using a random‐effect model.

**Results:**

Thirteen studies (3 in vivo and 10 in vitro) met inclusion criteria, displaying methodological heterogeneity (8 analyzing surface, 3 linear, 8 angular, and 3 interimplant distance deviations). The studies evaluated seven IOS (Aoralscan 3, Carestream 3600, iTero Element 2, iTero Element 5D, Primescan, Trios 3, and Trios 4) and two SPG devices (PIC and ICam4D). The number of implants ranged from 4 to 8. SPG reported higher accuracy than IOS in 10 of 13 studies. One in vitro study found IOS to have higher trueness but lower precision, another in vitro study found higher accuracy with IOS, and one in vivo study showed comparable trueness. Meta‐analyses of in vitro studies revealed significant differences favoring SPG in surface deviation trueness and precision, and angular deviation trueness (*p* < 0.05), with reported effects of 3.426, 4.893, and 1.199. SPG showed surface trueness and precision, and angular trueness mean ranges 5.18–48.74 and 0.10–5.46 μm, and 0.24°–0.80°, while IOS ranges 14.8–67.72 and 3.90–37.07 μm, and 0.28°–1.74°.

**Conclusions:**

Within study limitations, SPG showed to be a more reliable technology than IOS for complete‐arch digital implant impression, exhibiting significantly greater trueness and precision. IOS reported an angular deviation exceeding the 1° threshold required for a passive fit. Further clinical trials are required for conclusive evidence. Until then, a rigid prototype try‐in is still recommended.

**Trial Registration:** CRD42024490844

## 1 Introduction

To deliver a passive fit screw‐retained complete arch implant‐supported fixed dental prostheses (FDP), an accurate implant impression is mandatory [1]. The capability of recording the implant coordinates is defined by accuracy, composed of trueness and precision (ISO5725–1). Trueness defines the conformity of measurements to actual values, and precision refers to the consistency of multiple repeated measurements [2]. Continuous technology improvements have made digital implant impressions a valid alternative to conventional impressions in terms of accuracy and practicality [1, 3, 4]. Moreover, digital impression was advocated to increase patient comfort, avoid the need for pouring and casting laboratory procedures, and facilitate direct application of computer‐aided design and computer‐aided manufacturing (CAD‐CAM) additive and subtractive technologies and the related materials such as titanium, zirconia, and polymethylmethacrylate (PMMA) [5, 6, 7].

Nowadays, two digital technologies are available in the global market to record complete‐arch implant impressions: intraoral optical surface scan (IOS) and extraoral stereophotogrammetry (SPG) [8, 9]. The accuracy of IOS has been proven to be reliable for single crowns and short‐span FDPs [10]. However, the IOS three‐dimensional (3D) reconstruction algorithm, which acquires and stitches consecutive 3D images, is less efficient and potentially inaccurate in case of clinical scenarios with unstable reference points, long‐span edentulous ridges, or completely edentulous arches. Therefore, the main limitation of IOS is intrinsic to its intraoral scanning use, and several factors have been shown to affect its overall performance [11, 12, 13, 14]. For these reasons, the feasibility of IOS for complete‐arch implant impression is still considered controversial, with the lower jaw experiencing the worst results, although the application of artificial landmarks seems to enhance its use [11, 13, 14].

SPG digital impressions are based on an extraoral device featuring two stereo‐cameras that simultaneously identify and capture the 3D positions of fiducial optical geometries on specific scan bodies (SBs) and their spatial relationships [15]. SPG detects only implant coordinates, whereas intraoral dental and soft tissue anatomy cannot be recorded [16, 17]. The absence of a stitching process contemplated in SPG technology, combined with the extraoral recording, suggests a potential clinical application of SPG as a more accurate alternative to IOS for complete‐arch implant digital impression.

In recent years, several studies have compared the accuracy of IOS and SPG for complete‐arch implant impressions. However, differences in the choice of reference data (coordinate measuring machine (CMM), industrial optical scanner, and desktop optical laboratory scanner), accuracy outcomes (linear deviations, surface deviations as root mean square (RMS), and angular deviations), and scenarios (in vitro and in vivo) make it difficult to clearly compare the investigated technologies and reach conclusive results regarding the reliability of their clinical application.

The aim of the present systematic review and meta‐analysis was to assess and compare the accuracy of SPG versus IOS for implant complete‐arch digital impression in vivo, in vitro, or ex vivo, and to provide a high‐quality, valuable indication for clinicians interested in adopting these technologies in their practice.

## 2 Materials and Methods

### 2.1 Protocol and Registration

This review was registered at the International Prospective Register of Systematic Review (PROSPERO) of the National Institute of Health Research (Registration Number CRD42024490844). This review was reported according to the “Preferred Reporting Items for Systematic Reviews and Meta‐Analysis (PRISMA)” guidelines [18]. Ethical approval was not required for this systematic review.

### 2.2 Population, Intervention, Comparison, Outcomes, and Study Design

The focused question (Table 1) was defined as “In patients undergoing complete‐arch implant digital impression, what is the accuracy of intraoral scanning (IOS) compared to stereophotogrammetry (SPG)?” The question of the research was reported in PICOS (Population, Intervention, Comparison, Outcomes, and Study design) format:

**TABLE 1:** Inclusion criteria of studies into systematic review based on PICOS guides.

Population	Models/patients treated with dental implants
Intervention	Complete arch digital implant impression
Comparison	SPG versus IOS
Outcome	Accuracy (linear deviation, angular deviation, surface deviation, and interimplant distance) of SPG and IOS implant complete arch digital impression
Study design	Any studies with exception of case reports assessing in vivo, in vitro, or ex vivo the accuracy of SPG and IOS for complete arch implant impression

*P* = models/patient treated with dental implants;

I = complete arch digital implant impression;

C = SPG versus IOS;

O = accuracy (linear deviation, angular deviation, surface deviation, and inter‐implant distance) of SPG and IOS implant complete arch digital impression;

S = any studies with the exception of case reports assessing in vivo, in vitro, or ex vivo the accuracy of SPG and IOS for complete‐arch implant impression.

### 2.3 Inclusion Criteria

The inclusion and exclusion criteria were defined before the start of the study. To be included in the review, the studies had to be published in the English language and report on the accuracy of complete arch digital implant impressions recorded with SPG and IOS technologies and conducted with the proper sample size calculation. The research was limited to studies published after 2018 due to the novelty of the investigated technologies and their recent application to complete‐arch implant impressions. The main research subject had to evaluate the accuracy in terms of linear, angular, surface deviations, and inter‐implant distance between SPG and IOS. All the analyzed studies were published until May 20, 2024. Studies were not included if they reported the same data as later publications by the same authors; systematic reviews, commentaries, letters to the editor, case reports, and studies in animal models were also excluded. Relevant systematic reviews, as well as the reference lists of all included articles, were searched by hand to identify further publications.

### 2.4 Search Strategy

Electronic research was performed involving different databases (MEDLINE, PubMed, Embase, and Cochrane Library) without any limitation for publication year. The electronic search syntax in MEDLINE and PubMed, Embase, and Cochrane CENTRAL were: (“Photogrammetry”[MeSH Terms] OR “stereophotogrammetry”[All Fields]) AND (“implant dentistry”[All Fields] OR “Dental Prosthesis”[MeSH Terms]) AND (“Implant digital impression”[All Fields] OR “Intraoral scanner”[All Fields] OR “Intraoral scanners”[All Fields]) AND (“complete arch”[All Fields] OR “full‐arch”[All Fields] OR “edentulous”[All Fields]) AND (“Accuracy”[All Fields] OR “trueness”[All Fields] OR “precision”[All Fields]) and Scopus (“Photogrammetry” OR “stereophotogrammetry” AND “implant dentistry” OR “Dental Prosthesis” AND “Implant digital impression” OR “Intraoral scanner” OR “Intraoralscanners” AND “complete‐arch” OR “full‐arch” OR “edentulous” AND “Accuracy” OR “trueness” OR “precision”), in title/abstract/keywords, respectively. Moreover, bibliographies of relevant systematic reviews were analyzed to cross‐check the data. Additionally, a manual search of the reference lists of included studies and examinations of meeting abstracts related to the PICOS question was executed. The decision criteria for including or excluding the studies are shown in Table 2.

**TABLE 2:** Inclusion and exclusion criteria.

Inclusion criteria	Exclusion criteria
English literature	Expert opinions, case reports, case series, reviews
Peer‐reviewed in vitro, ex vivo, and in vivo articles published from 2018	Articles reporting on accuracy of impression for tooth‐supported or removable prostheses
Minimum sample size: 10 test scans per device in vitro and ex vivo, 1 scan per device in vivo	Only qualitative evaluation of impression and/or model accuracy
Intraoral scanner and stereophotogrammetry groups available in the study	Digital impression devices not available on the market
Mandibular and/or maxillary complete‐arch implant jaws/models	Patient‐reported outcomes studies
Accuracy studies assessing trueness and/or precision	

### 2.5 Study Selection

**FIGURE 1:**
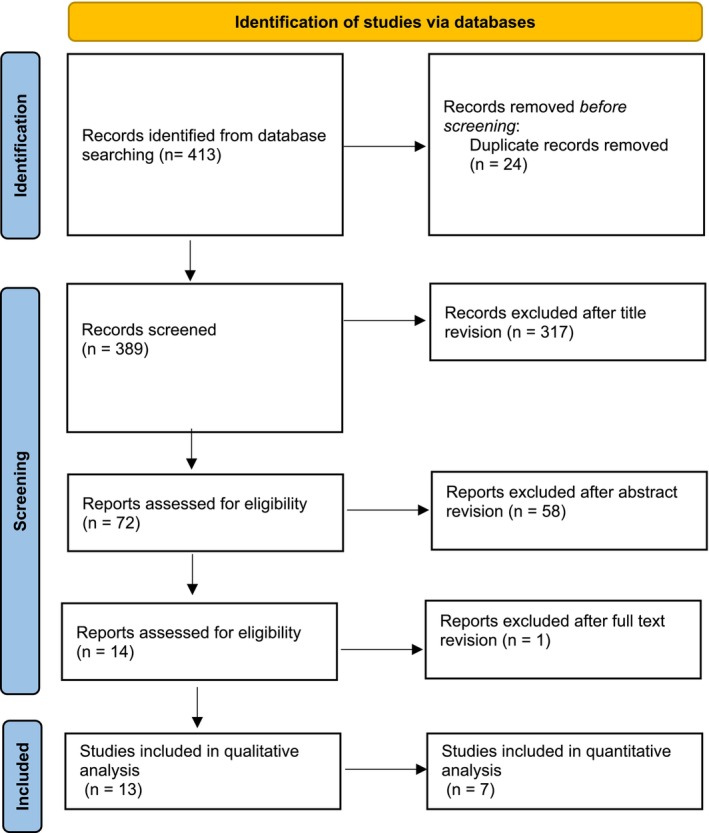
PRISMA flowchart of article selection.

All the gathered data were imported into a reference manager software program (Endnote X9, Clarivate Analytics, Philadelphia, PA) to automatically remove duplicates. Two reviewers (L.A. and P.C.) screened titles and abstracts independently, according to pre‐established criteria. Records lacking enough information in the title and abstract were kept for full‐text reading to be assessed for eligibility (Figure 1). The inter‐reviewer reliability (kappa correlation coefficient) of the title/abstract and full‐text screening was 0.86% and 99.27%, respectively, and disagreements were resolved by consensus.

### 2.6 Data Extraction

Data of all included studies were independently extracted by the two reviewers, then double checked, and any disagreement was discussed within the group with a third reviewer (A.P.) According to the PICOS question, the following data were extracted from the selected articles: author(s) and year of publication, study type, number/type of implants and jaw, compared impression types, investigated IOS/SPG devices, test scans number per device, reference scan device, scan body type, accuracy outcomes, trueness results and/or precision results, and conclusions.

### 2.7 Risk of Bias

**FIGURE 2:**
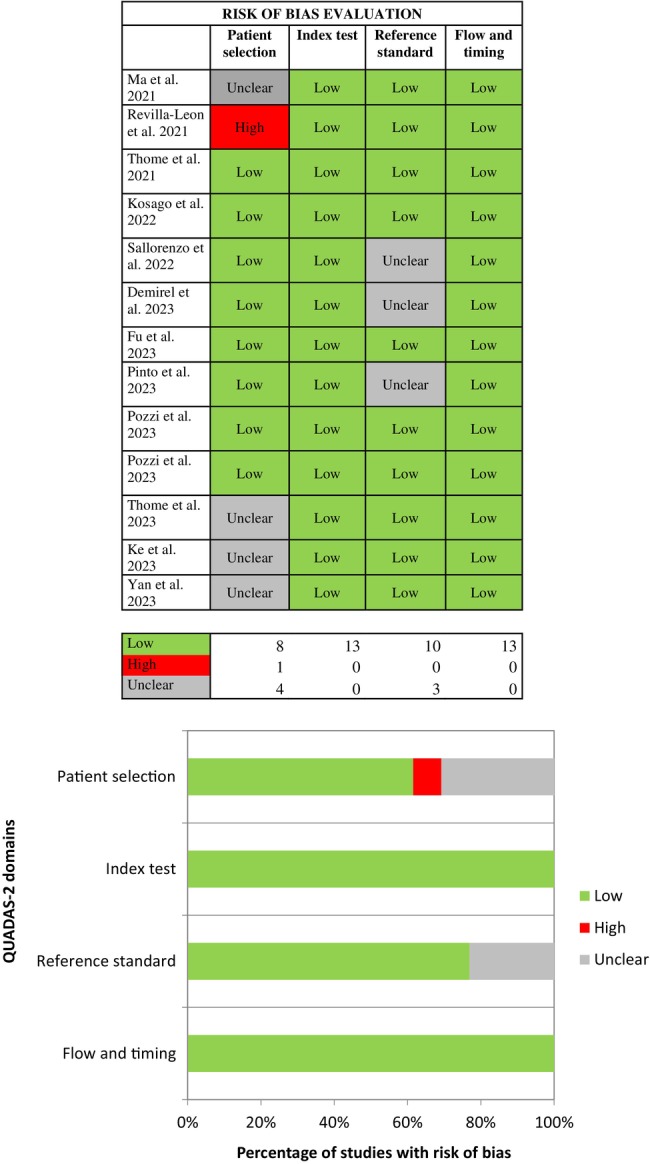
Risk of bias evaluation (QUADAS‐2).

The quality of the included studies was assessed independently by two authors (L.A. and P. C.) using the Quality Assessment of Diagnostic Accuracy Studies checklist (QUADAS‐2) reporting the risk of bias (ROB) of the included studies [19]. Any disagreement between the two reviewers was solved by consulting the third reviewer (A.P.). This tool consists of four key domains for assessment: patient selection, index test, reference standard, and flow and timing. The tool questions of each domain were adapted to the present review as follows: Domain 1: Was a sample size calculation executed? Domain 2: Were the types of intraoral scanning and stereophotogrammetry devices described? Were the types of software for the accuracy assessment described? Domain 3: Was the device used as a reference specified? Was the accuracy of the reference measuring device specified? Domain 4: Was the reference the same for all the measurements? The applicability evaluation tool was not adopted as it was not fit for the present systematic review (Figure 2).

### 2.8 Statistical Analysis

Three meta‐analyses were performed to check for significant differences in the angular deviation values in terms of trueness, and in the surface deviation values in terms of trueness and precision experienced by IOS and SPG. Meta‐analyses were performed using the random‐effects model. Cochran's Q‐statistic and the *I* ^*2*^ statistic were used to calculate heterogeneity between the included studies. *p* < 0.05 were considered statistically significant. A minimum of three studies were needed to perform meta‐analysis. For all meta‐analyses, the Cohen's D was the chosen effect size. Due to the limited number of studies included in each of the three meta‐analyses, publication bias tests were not performed. The software version 28 of SPSS (IBM, Armonk, NY, USA) was used.

## 3 Results

Electronic and manual searches identified 413 titles (Figure 1). After duplicate removal and title revision, 341 studies were excluded, leading to 72 remaining abstracts. After abstract and full‐text reading, 59 full‐text articles were excluded, leading to 13 articles included for qualitative analysis. Details of the 13 included studies are reported in Table 3. Seven in vitro studies were included in quantitative analysis, three analyzing surface, two angular, and two surface and angular deviations. Six studies were excluded from quantitative analysis due to the heterogeneity of the reported outcomes. Ten studies were in vitro [20, 21, 22, 23, 24, 25, 26, 27, 28, 29], of which six were on maxilla [20, 21, 22, 24, 26, 29] and four on mandible [23, 25, 27, 28] models, and three in vivo [30, 31, 32] on both mandible and maxilla. Included studies study design details are reported in Table 4.

**TABLE 3:** Main outcomes of included studies.

Author, year	Study type	Number/type of implants and jaw	Impression types compared	IOS/SPG device	Test scan number per device	Reference scan	Scan body type	Outcomes	Results trueness	Results precision	Conclusions
Ma et al., 2021	In vitro	6 (RC, Institute Straumann AG), maxilla	‐IOS ‐SPG ‐CI	‐TRIOS 3, 3Shape ‐ICam4D, Imetric4D	10	EOS (E4, 3Shape); 4 μm accuracy	‐CARES Mono Scanbody, Institute Straumann AG ‐ICamBody, Imetric4D	Surface deviation (RMS)	‐IOS mean 43.78 ± 4.03 μm ‐SPG mean 24.43 ± 0.35 μm	‐IOS mean 37.07 ± 3.98 μm ‐SPG mean 2.32 ± 0.85 μm	SPG obtained the lowest and IOS the highest 3D discrepancy in terms of trueness and precision.
Revilla‐León et al., 2021	In vitro	6 (RC, Institute Straumann AG), maxilla	‐IOS (2) ‐SPG ‐CI	‐iTero Element, Cadent (IOS1) ‐TRIOS 3, 3Shape A/S (IOS2) ‐ICam4D, Imetric4D	10	CMM (Contura G2, Carl Zeiss Industrielle Messtechnik GmbH); 1 μm accuracy	‐CARES Mono Scanbody, Institute Straumann AG ‐ICamBody, Imetric4D	‐Linear deviation (X, Y, Z, 3D) ‐Angular deviation (XZ, YZ)	‐IOS1 median (4.1, 17.5, −4.1, 18.4 μm; −0.1°, −0.1°) ‐IOS2 median (9.7, 18.0, −4.9, 21.1 μm; −0.2°, 0.2°) ‐SPG median (23.8, 73.7, −4.7, 77.6 μm, 0.1°, 0.3°)	‐IOS1 IQR (16.6, 48.9, 37.3 μm; 0.1°, 0.3°) ‐IOS2 IQR (54.6, 54.9, 20.7 μm; 0.4°, 0.4°) ‐SPG IQR (308.7, 273.6, 27.2 μm; 0.6°, 0.6°)	SPG showed higher deviations compared to IOS1 and IOS2.
Tohme et al., 2021	In vitro	4 (RC, Institute Straumann AG), maxilla	‐IOS ‐SPG	‐TRIOS 3, 3Shape A/S ‐PiC Camera, PiC Dental	15	EOS (E3, 3Shape); 7 μm accuracy	‐CARES Mono Scanbody, Institute Straumann AG ‐PiCabutment, PiC dental	‐Surface deviation (RMS) ‐Angular deviation	‐IOS mean 0.536 ± 0.063 μm; 1.744° ± 0.175° ‐SPG mean 0.078 ± 0.001 μm; 0.724° ± 0.064°	‐IOS mean 0.039 ± 0.009 μm ‐SPG mean 0.014 ± 0.013 μm	SPG showed higher trueness and similar angular precision compared to IOS
Kosago et al., 2022	In vitro	5 (RC BLT Institute Straumann AG), mandible	‐IOS (3) ‐SPG ‐CI	‐iTero Element 2, Align Technology (IOS1) ‐Primescan, Dentsply‐Sirona (IOS2) ‐TRIOS 4, 3Shape A/S (IOS3) ‐PiC Camera, PiC Dental	10	EOS (E4, 3Shape); 4 μm accuracy	‐CARES Mono Scanbody, Institute Straumann AG ‐PiCabutment, PiC dental	Surface deviation (RMS)	‐IOS1 mean 67.72 ± 7.18 μm ‐IOS2 mean 57.24 ± 2.05 μm ‐IOS3 mean 52.14 ± 3.88 μm ‐SPG mean 48.74 ± 1.80 μm	‐IOS1 mean 36.84 ± 12.64 μm ‐IOS2 mean 28.58 ± 8.03 μm ‐IOS3 mean 19.39 ± 3.61 μm ‐SPG mean 5.46 ± 1.10 μm	SPG showed highest accuracy. IOS1 showed lowest accuracy.
Sallorenzo et al., 2022	In vitro	6 (Implant Protesis Dental 2004 SL), 2 maxillae	‐IOS ‐SPG	‐TRIOS 3, 3Shape A/S (IOS1) ‐PiC Camera, PiC Dental	20 (10 per arch)	CMM (Global Evo 09.15.08, Serial No. 906; Hexagon Manufacturing Intelligence)	‐Elos Accurate, Elos Medtech ‐PiCabutment, PiC dental	‐Distance deviation ‐Angular deviation	‐IOS mean 100 μm ‐SPG mean 20 μm ‐IOS mean 1.177° ‐SPG mean 0.354°	‐IOS SD ±292 μm ‐SPG SD ±32 μm ‐IOS SD ±0.474° ‐SPG SD ±0.280°	SPG delivered more accurate values than IOS, particularly in terms of precision.
Demirel et al., 2023	In vitro	6 (MUA, NobelBiocare AB), mandible	‐IOS (2) ‐SPG ‐CI + EOS (2)	‐Primescan, Dentsply‐Sirona (IOS1) ‐TRIOS 4, 3Shape A/S (IOS2) ‐ICam4D, Imetric4D	10	Industrial optical scanner (ATOS Core 80 5MP; GOM)	‐Elos Accurate, Elos Medtech ‐ICamBody, Imetric4D	‐Surface deviation (RMS) ‐Angular deviation	‐IOS1 median 51 μm; 0.34° ‐IOS2 median 58.5 μm; 0.48° ‐SPG median 104 μm; 0.52°	‐IOS1 median 5.80 μm; 0.03° ‐IOS2 median 12.90 μm; 0.16° ‐SPG median 1.50 μm; 0.06°	IOSs showed higher trueness and lower or comparable precision compared to SPG
Fu et al., 2023	In vivo	4–6 (MUA, NobelBiocare AB), 9 maxilla, and 16 mandible (15 patients)	‐IOS ‐SPG	‐TRIOS 3, 3Shape A/S ‐PiC Camera, PiC Dental	1 per arch	Open tray splinted CI (polyvinyl siloxane [Silagum putty/light], DMG) +EOS (T8, Medit Corp); 4 μm accuracy	‐ Bridge+, Segma (SBs with prefabricated aids) ‐PiCabutment, PiC dental	‐Surface deviation (RMS) ‐Distance deviation ‐Angular deviation	‐IOS median 69 μm; median 75 μm; 0.40° ‐SPG median 45 μm; 60 μm; 0.31°	N/A	Accuracy of SPG and IOS with prefabricated scan aid was clinically comparable
Ke et al., 2023	In vitro	6 (MUA, NobelBiocare AB), maxilla	‐IOS ‐SPG ‐SSLS	‐Aoralscan 3, Shining 3D ‐ICam4D, Imetric4D	10	EOS (T710, Medit) 2.9 μm	‐NB MU‐R SB, Segma ‐ICamBody, Imetric4D	Distance deviation	‐IOS mean 214.6 ± 96.0 μm ‐SPG mean 58.4 ± 3.1 μm	‐IOS mean 329.1 ± 328.8 μm ‐SPG mean 6.8 ± 6.0 μm	SPG showed higher accuracy compared to IOS
Pinto et al., 2023	In vitro	4–6 (SRA RC, Institute Straumann AG), mandible	‐IOS (2) ‐SPG (2) ‐EOS	‐iTero Element 5D, Align Technology (IOS1) ‐TRIOS 3, 3Shape A/S (IOS2) ‐ ICam4D, Imetric4D (SPG1) ‐PiC Camera, PiC Dental (SPG2)	12 per arch	Industrial optical scanner (ATOS Capsule Scanner; GOM GmbH)	‐Straumann CARES; Institut Straumann AG ‐ICamBody, Imetric4D ‐PiCabutment, PiC dental	Surface deviation (RMS)	4 implants: ‐IOS1 mean 20.50 μm (17.37;23.63) ‐IOS2 mean 20.52 μm (18.33;22.72) ‐SPG1 mean 7.01 μm (6.11;7.91) ‐SPG2 mean 5.18 μm (4.60;5.76) 6 implants: ‐IOS1 mean 38.86 μm (34.01;43.71) ‐IOS2 mean 40.32 μm (36.29;44.36) ‐SPG1 mean 8.67 μm (8.06;9.28) ‐SPG2 mean 13.88 μm (12.62;15.14)	N/A	SPG showed higher trueness compared to IOS
Pozzi et al., 2023	In vivo	4–6 (MUA, NobelBiocare AB), 5 maxilla, and 6 mandibles (11 patients)	‐IOS ‐SPG	‐TRIOS 4, 3Shape A/S ‐PiC Camera, PiC Dental	1 per arch	Open tray CI (plaster, SnowWhite Plaster no. 2; Kerr) +EOS (D2000; 3Shape); 5 μm accuracy	‐Elos Accurate, Elos MedTech ‐PiCabutment, PiC dental	‐Linear deviation (X, Y, Z, 3D) ‐Angular deviation	‐IOS mean −19.8 μm, −4.1, −41.9, 137.2 μm; 0.79° ‐SPG mean −24.8, −3.4, −20.9, 87.6 μm; 0.38°	‐IOS SD ‐ ± 110.2 μm ‐ ± 44.3 μm ‐ ± 127.5 μm ‐ ± 115.5 μm ‐ ± 0.59° ‐SPG SD ‐ ± 71.8 μm ‐ ± 29 μm ‐ ± 79.1 μm ‐ ± 74.2 μm ‐ ± 0.29°	SPG showed higher accuracy compared to IOS
Pozzi et al., 2023	In vitro	4 (MUA, NobelBiocare AB), mandible	‐IOS ‐SPG	‐TRIOS 4, 3Shape A/S ‐PiC Camera, PiC Dental	30	EOS (D2000; 3Shape) 5 μm accuracy	‐Elos Accurate, Elos Medtech ‐PiCabutment, PiC dental	‐Linear deviation (X, Y, Z, 3D) ‐Angular deviation	‐IOS 5.21, 2.03, −1.85, 52.81 μm; 0.28° ‐SPG 12.81, 0.95, 20.78, 33.42 μm; 0.24°	‐IOS SD ± 50.51 μm ± 14.54 μm ± 37.31 μm ± 37.11 μm ± 0.14° ‐SPG SD ± 19.23 μm ± 7.15 μm ± 20.42 μm ± 17.71 μm ± 0.04°	SPG showed higher accuracy compared to IOS
Tohme et al., 2023	In vitro	4 (SRA RC, Institute Straumann AG), maxilla	‐IOS ‐SPG ‐CI	‐TRIOS 4, 3Shape A/S ‐PiC Camera, PiC Dental	15	EOS (E3; 3Shape A/S) 7 μm accuracy	‐Straumann CARES; Institut Straumann AG ‐PiCabutment, PiC dental	‐Surface deviation (RMS) ‐Angular deviation	‐IOS mean 0.148 ± 0.061 μm; 1.081° ± 0.348° ‐SPG mean 0.088 ± 0.006 μm; 0.809° ± 0.005°	‐IOS mean 0.090 ± 0.110 μm; 0.221° ± 0.088° ‐SPG mean 0.001 ± 0.001 μm; 0.010° ± 0.011°	SPG showed higher accuracy compared to IOS
Yan et al., 2023	In vivo	4–6–8 (MUA, Straumann), 5 maxillae and 6 mandibles (17 patients)	‐IOS ‐SPG	‐CS3600; Carestream ‐ICam4D, Imetric4D	1 per arch	Open‐tray splinted CI (polyether [Impregum Penta Soft, 3 M ESPE]) + EOS (E4; 3Shape A/S); 4 μm accuracy	‐Straumann CARES; Institut Straumann AG or CAD‐CAM SBs (SYSTEM TI 5‐BLANK; Adentatec GmbH) ‐ICamBody, Imetric4D	‐Surface deviation (RMS)	‐IOS median 48.95 μm ‐SPG median 17.00 μm	N/A	SPG showed higher accuracy compared to IOS

Abbreviations: 3D, three‐dimensional; BLT, bone level tapered; CC, conical connection; CI, conventional impression; CMM, coordinate measuring machine; EOS, extraoral scanner; IOS, intraoral scanner; IQR, interquartile range; MUA, multiunit abutment; RC, regular connection; RC, regular platform; RMS, root mean square; SB, scan body; SD, standard deviation; SPG, stereophotogrammetry; SRA, screw‐retained abutment; SSLS, self‐developed structured light scanning.

**TABLE 4:** Selected articles details.

Variables	(Number of articles)
Number of implants per model/jaw	4 (3), 5 (1), 6 (5), 4–6 (3), 4–6‐8 (1)
Type of impressions compared	IOS vs. SPG (6), CI vs. IOS vs. SPG (2), CI vs. 2IOS vs. SPG (1), CI vs. 3IOS vs. SPG (1), CI + 2EOS vs. 2IOS vs. SPG (1), IOS vs. SSLS vs. SPG (1), 2IOS vs. 2SPG vs. EOS (1)
IOS device	Trios3 (6), Trios4 (5), iTero Element 5D (2), Primescan (2), Aoralscan 3 (1), CS3600 (1), iTero Element (1)
SPG device	PicCamera (8), ICam4D (6)
Reference	EOS (6), CMM (2), Industrial optical scanner (1), CI + EOS (3)
IOS SB	CaresMono (7), Elos Accurate (4), Bridge+ (1), NBMU‐R (1), CAD‐CAM produced (1)
SPG SB	Pic flag (8), ICam body (6)

Abbreviations: CI, conventional impression; CMM, coordinate measuring machine; EOS, extraoral scanner; IOS, intraoral scanner; SB, scan body; SPG, stereophotogrammetry; SSLS, self‐developed structured light scanning.

The most adopted accuracy outcomes were angular (*n* = 8) [21, 22, 24, 25, 28, 29, 30, 31] and surface deviation (RMS) (*n* = 8) [20, 22, 23, 25, 27, 29, 30, 32], followed by linear (*n* = 3) [21, 28, 31] and distance deviation (*n* = 3) [24, 26, 30]. Eight studies considered more than one accuracy outcome [21, 22, 24, 25, 28, 29, 30, 31], while five considered just one accuracy outcome [20, 23, 26, 27, 32].

Four in vitro studies were included in quantitative analysis considering trueness angular deviations [22, 24, 28, 29], five in vitro studies considering trueness surface deviations (RMS) [20, 22, 23, 27, 29], and four in vitro studies considering precision surface deviations [20, 22, 23, 27, 29].

The test for heterogeneity revealed an overall Q‐statistic index of 70 056 (RMS trueness), 221 852 (angular trueness), and 42 869 (RMS precision), and an *I* ^*2*^ statistic index of 96.7% (RMS trueness), 99.7% (angular trueness), and 98.0% (RMS precision).

SPG showed higher accuracy compared to IOS in 10 studies [20, 22, 23, 24, 26, 27, 28, 29, 31, 32] out of 13. IOS showed higher trueness and lower precision in one in vitro study [25], higher accuracy in one in vitro study [21], and comparable trueness results were detected by one in vivo study [30].

### 3.1 Distance Deviation

Three studies (*n* = 3) reported as accuracy outcomes the distance deviations between the SBs of the test scans compared to the reference scan [24, 26, 30].

Two in vitro studies reported higher accuracy for SPG with respect to IOS [24, 26], with Sallorenzo et al. reporting mean distance deviation (trueness values with SD considered as precision) of 20 ± 32 μm (SPG) versus 100 ± 292 μm (IOS). Ke et al. reported mean distance deviation according to trueness (58.4 ± 3.1 μm (SPG) vs. 214.6 ± 96.0 μm (IOS)) and precision (6.8 ± 6.0 μm (SPG) vs. 329.1 ± 328.8 μm (IOS)).

One in vivo study reported comparable trueness results, reporting median distance deviations of 75 μm (SPG) versus 60 μm (IOS with prefabricated aids) [30], without contemplating precision.

### 3.2 Linear Deviation

Three studies (*n* = 3) reported as accuracy outcomes the linear deviations on the X, Y, and Z axes and 3D deviation intended as Euclidean distance between the centroids of the implant platforms of the test scans compared to the reference scan [21, 28, 31].

Two studies (one in vitro and one in vivo) reported higher accuracy by SPG compared to IOS [28, 31]. Pozzi et al. reported in vitro reporting mean X, Y, Z, and 3D deviations (trueness values with SD considered as precision) of 12.81 ± 19.23, 0.95 ± 7.15, 20.78 ± 20.42, and 33.42 ± 17.71 μm for SPG versus 5.21 ± 50.51, 2.03 ± 14.54, −1.85 ± 37.31, and 52.81 ± 37.11 μm for IOS. In vivo, Pozzi et al. reported mean X, Y, Z, and 3D deviations of 24.8 ± 71.8, −3.4 ± 29, −20.9 ± 79.1, and 87.6‐ ± 74.2 μm for SPG versus 19.8 ± 110.2, −4.1 ± 44.3, −41.9 ± 127.5, and 137.2 ± 115.5 μm for IOS.

One in vitro study reported lower accuracy for SPG with respect to IOS [21], reporting median X, Y, Z, and 3D deviations in terms of trueness of 23.8, 73.7, −4.7, and 77.6 μm for SPG versus 4.1, 17.5, −4.1, and 18.4 μm for IOS1, and 9.7, 18.0, −4.9, and 21.1 μm for IOS2. Precision according to interquartile range (IQR) was 308.7, 273.6, and 27.2 μm for SPG versus 16.6, 48.9, and 37.3 μm for IOS1, and 54.6, 54.9, and 20.7 μm for IOS2 without considering 3D deviation in terms of precision.

### 3.3 Angular Deviation

Eight studies (*n* = 8) reported as accuracy outcomes the angular deviations between the implant axes of the test scans compared to the reference scan [21, 22, 24, 25, 28, 29, 30, 31].

Six studies (four in vitro and two in vivo) reported higher accuracy for SPG relative to IOS [22, 24, 28, 29, 30, 31].

Considering in vitro studies, Thome et al. reported mean angular deviation values (trueness) of 0.724° ± 0.064° (SPG) versus 1.744° ± 0.175° (IOS), without specifying precision mean values [22]; Sallorenzo et al. reported mean angular deviation values (trueness values with SD considered as precision) of 0.354° ± 0.280° (SPG) versus 1.177° ± 0.474° (IOS) [24]. Pozzi et al. reported mean angular deviation values (trueness values with SD considered as precision) of 0.24° ± 0.04° (SPG) versus 0.28° ± 0.14° (IOS) [28]; Thome et al. reported mean angular deviation values in terms of trueness of 0.809° ± 0.005° (SPG) versus 1.081° ± 0.348° (IOS), and precision of 0.010° ± 0.011° (SPG) versus 0.221° ± 0.088° (IOS) [29].

Considering in vivo studies, Fu et al. reported median deviation values only in terms of trueness of 0.31° (SPG) versus 0.40° (IOS) [30] and Pozzi et al. reported mean deviation values (trueness values with SD considered as precision) of 0.38° ± 0.29° (SPG) versus 0.79° ± 0.59° (IOS) [31].

Two in vitro studies reported lower accuracy of SPG compared to IOS [21, 25]. Revilla‐León et al. reported median values of angular deviation (XZ, YZ) in terms of trueness of −0.3° (SPG) versus 0.1° (IOS1) 0.2° (IOS2) and IQR in terms of precision of 0.6° (SPG) versus 0.3° (IOS 1) and 0.4° (IOS2) [21]. Demirel et al. reported median values of angular deviation in terms of trueness of 0.52° (SPG) versus 0.34° (IOS1) and 0.48° (IOS2) and precision of 0.6° (SPG) versus 0.3° (IOS1) 0.4° (IOS2) [25].

**FIGURE 3:**
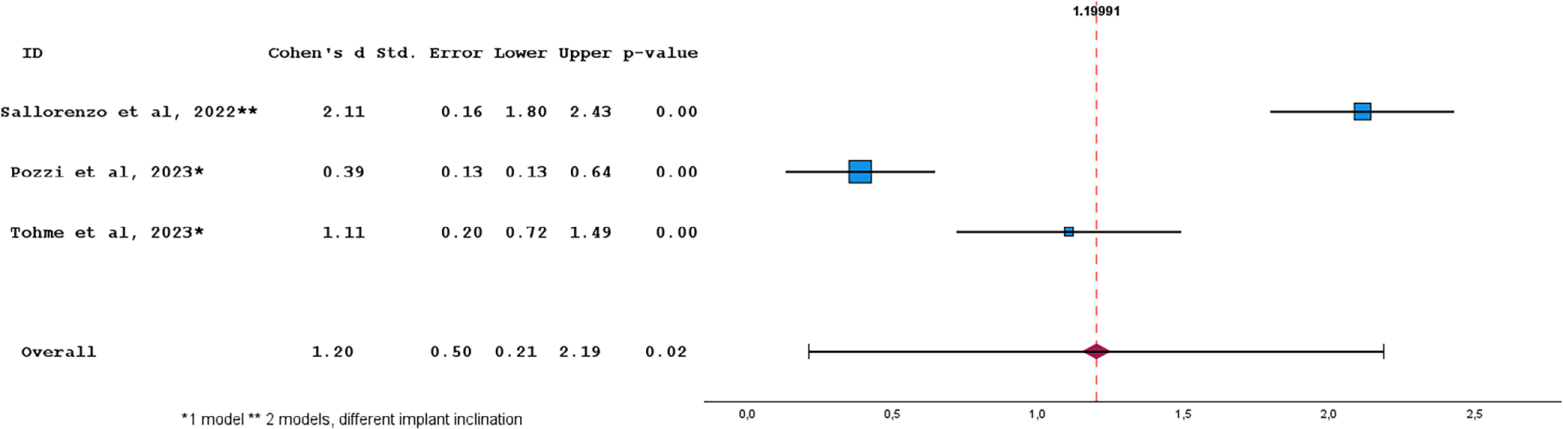
Random‐effects meta‐analysis for angular deviation trueness studies between Photogrammetry vs intraoral scanning. The squares represent the effect sizes for each study, while the horizontal lines indicate the 95% confidence intervals. The dotted line and diamond represent the overall effect size. Homogeneity: Q = 69.34; df = 2; *p*‐value < 0.001; *I* ^2^ = 0.97.

Four in vitro studies were included in quantitative analysis considering trueness of angular deviations [22, 24, 28, 29]. A homogeneity test was executed and resulted in statistically significant outcome (*p* < 0.001) with a heterogeneity index (*I* ^*2*^) of 99.7%. The overall effect size was not statistically significant despite a high effect size of 2.809. A sensitivity analysis was performed to test the robustness of the meta‐analysis. All four included studies showed significant effects. However, one outlier with a large effect size and high standard error skewed the results (*p* = 0.09). Excluding this study, “Thome et al. 2021” led to a significant overall effect (*p* < 0.05), reducing heterogeneity. The revised meta‐analysis confirmed the absence of publication bias (*p* = 0.929) and justified using a random‐effects model (Q (2) = 69.342, *p* < 0.001; *I* ^*2*^ = 96.6%). Ultimately, the meta‐analysis indicated a significant difference in angular deviation trueness between IOS and SPG (Cohen's D = 1.20, *p* = 0.02), in favor of SPG, as shown in the forest plot (Figure 3).

### 3.4 Surface Deviation (RMS)

Eight studies (*n* = 8) reported accuracy outcomes in the surface deviation in terms of RMS between the SB/implant surfaces of the test scans compared to the ones of the reference scan [20, 22, 23, 25, 27, 29, 30, 32].

Seven studies (five in vitro and two in vivo) reported higher accuracy for SPG compared to IOS [20, 22, 23, 27, 29, 30, 32].

Considering in vitro studies, Ma et al. reported mean RMS deviation values in terms of trueness of 24.43 ± 0.35 μm (SPG) versus 43.78 ± 4.03 μm (IOS) and precision of 2.32 ± 0.85 μm (SPG) versus 37.07 ± 3.98 μm (IOS) [20]; Thome et al. reported mean RMS deviation values in terms of trueness of 7.80 ± 0.10 μm (SPG) versus 53.60 ± 6.30 μm (IOS) and precision of 1.40 ± 1.30 μm (SPG) versus 3.90 ± 0.90 μm (IOS) [22]; Kosago et al. reported mean RMS deviation values in terms of trueness of 48.74 ± 1.80 μm (SPG) versus 67.72 ± 7.18 μm (IOS1), 57.24 ± 2.05 μm (IOS2), and 52.14 ± 3.88 μm (IOS3) and precision of 5.46 ± 1.10 μm (SPG) versus 36.84 ± 12.64 μm (IOS1), 28.58 ± 8.03 μm (IOS2), and 19.39 ± 3.61 μm (IOS3) [23]; Pinto et al. reported mean RMS deviation values in terms of trueness on four implants of 7.01 μm (6.11;7.91) (SPG 1) mean 5.18 μm (4.60;5.76) (SPG2) versus mean 20.50 μm (17.37;23.63) (IOS1) and 20.52 μm (18.33;22.72) (IOS2), and on six implants of 8.67 μm (8.06;9.28) (SPG1), mean 13.88 μm (12.62;15.14) (SPG 2) versus mean 38.86 μm (34.01;43.71) (IOS1) and mean 40.32 μm (36.29;44.36) (IOS2), without reporting data on precision [27]; Thome et al. reported mean RMS deviation values in terms of trueness of 8.80 ± 0.60 μm (SPG) versus mean 14.8 ± 6.1 μm (IOS) and precision of 0.1 ± 0.1 μm (SPG) versus mean 9.0 ± 11.0 μm (IOS) [29].

Considering in vivo studies, Fu et al. reported median RMS deviation values in terms of trueness of 45 μm (SPG) versus 69 μm (IOS), without reporting data on precision [30], and Yan et al. reported median RMS deviation values in terms of trueness of 17.00 μm (SPG) versus 48.95 μm (IOS), without reporting data on precision [32].

One in vitro study reported lower trueness for SPG with respect to IOS, with median RMS deviation values of 104 μm (SPG) versus 51 μm (IOS1) and 58.5 μm (IOS2), but higher precision for SPG compared to IOS, with median RMS deviation values of 1.50 μm (SPG) versus median 5.80 μm (IOS1) and median 12.90 μm (IOS2) [25].

**FIGURE 4:**
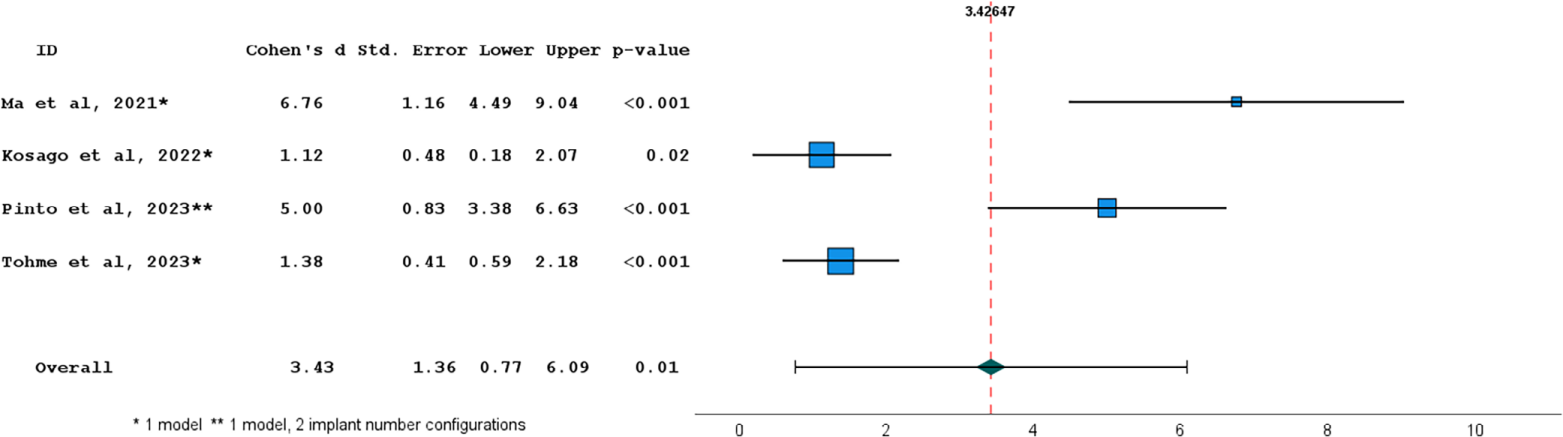
Random‐effects meta‐analysis for surface deviation trueness studies between Photogrammetry vs intraoral scanning. The squares represent the effect sizes for each study, while the horizontal lines indicate the 95% confidence intervals. The dotted line and diamond represent the overall effect size. Homogeneity: Q = 35.60; df = 2; *p*‐value < 0.001; *I* ^2^ = 0.95.

Five in vitro studies were included in quantitative analysis considering trueness surface deviation (RMS) [20, 22, 23, 27, 29]. A homogeneity test was executed and resulted in statistically significant (*p* < 0.001) with a heterogeneity index *I* ^*2*^ of 96.7%. It was necessary to remove the study of “Thome et al. 2021” from the meta‐analysis. The homogeneity test resulted in statistical significance (*p* < 0.001) with a heterogeneity index *I* ^*2*^ of 94.7%. The overall effect size resulted in a significant (*p* < 0.05), thus the null hypothesis of the absence of difference between IOS and SPG was refused. The effect between the two devices was 3.426 showing a great difference. The forest plot showed that for all the selected articles there was a significant difference between IOS and SPG (Figure 4).

**FIGURE 5:**
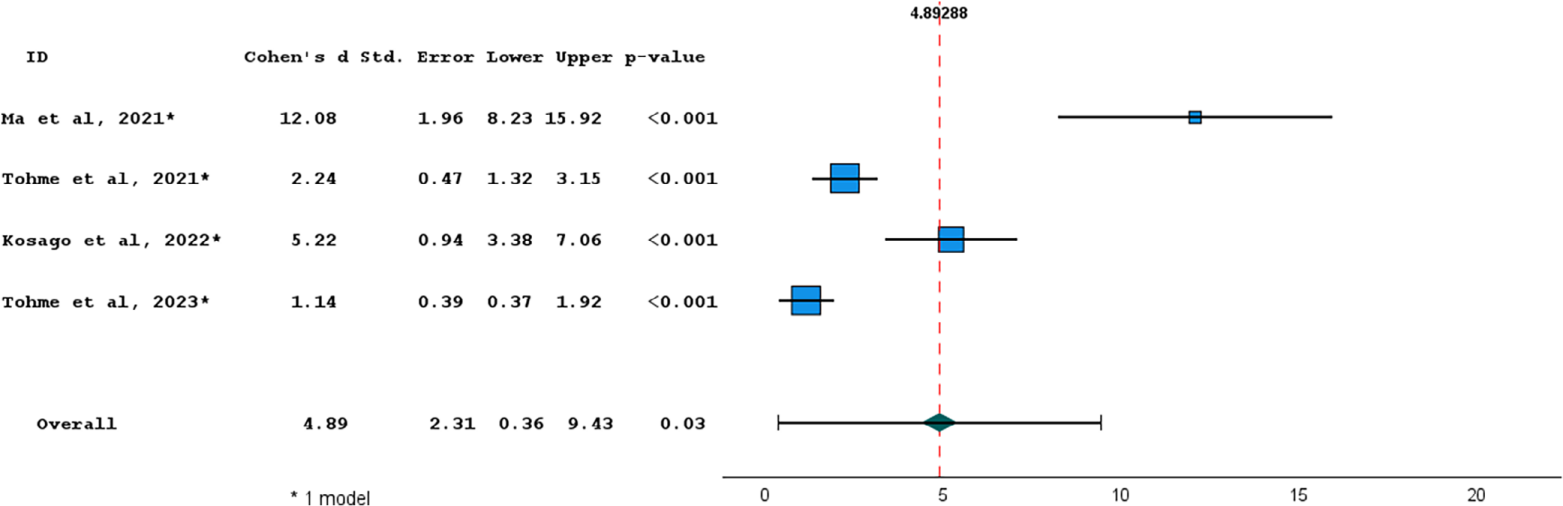
Random‐effects meta‐analysis for surface deviation precision studies between Photogrammetry vs intraoral scanning. The squares represent the effect sizes for each study, while the horizontal lines indicate the 95% confidence intervals. The dotted line and diamond represent the overall effect size. Homogeneity: Q = 42.87; df = 3; *p*‐value < 0.001; *I* ^2^ = 0.98.

Four in vitro studies were included in quantitative analysis considering precision surface deviation (RMS) [20, 22, 23, 27, 29]. A homogeneity test was executed and resulted in statistically significant (*p* < 0.001) with a heterogeneity index *I* ^*2*^ of 98.0%. The overall effect size resulted significant (*p* < 0.05); therefore, it was possible to refuse the null hypothesis of the absence of difference between IOS and SPG. The effect between the two devices was 4.893, showing a great difference. The forest plot showed that for all the selected articles there was a significant difference between IOS and SPG (Figure 5).

## 4 Discussion

Digital technologies are considered a viable alternative to conventional impressions for recording implant positions. Two different digital technologies (SPG and IOS) are available in the dental market and several recent studies have compared the accuracy of SPG and IOS. However, their application for complete‐arch digital implant impression remains controversial. The purpose of this systematic review and meta‐analysis was to assess and compare, in vitro, ex vivo, and in vivo, the accuracy of IOS versus SPG for complete‐arch implant impressions, presenting for the first time a quantitative analysis.

Thirteen studies (3 in vivo and 10 in vitro) met the inclusion criteria and were eligible for analysis. Three meta‐analyses were executed on angular deviations trueness, surface deviations trueness, and precision.

Generally, clinical accuracy investigations are subjected to different levels of criticality in the reference scan methodology and patient compliance. Even though only three clinical studies were included, in vivo trials needed indirect digitalization to obtain the reference scans (through the scan of the models). This method may provide a less accurate reference for the accuracy assessment compared to an in vitro study, where direct digitalization is possible with highly accurate technologies such as CMM, industrial optical scanners, and the latest generation of optical dental laboratory scanners. Furthermore, precision (intended as repeatability) assessment in vivo is complex to analyze, as each patient would need to undergo several scanning procedures, posing potential issues with patient compliance.

In the present systematic review, two out of three in vivo studies reported statistically significant higher trueness results for SPG compared to IOS [31, 32], with Pozzi et al. reporting mean IOS versus SPG 3D and angular deviation of 137.2 ± 115.5 μm versus 87.6 ± 74.2 μm and 0.79° ± 0.59° versus 0.38° ± 0.29°, and Yan et al. reporting IOS versus SPG RMS median deviation of 48.95 μm versus 17.00 μm. One study comparing SPG with IOS assisted by SBs with prefabricated auxiliary splinting geometry reported comparable trueness results, with median IOS versus SPG RMS and angular deviation of 69 μm versus 45 μm and 0.40° versus 0.31° [30]. Only one study indirectly reported in vivo precision considering the SD values [31]. Due to the low number and its heterogeneity of the in vivo studies, they were excluded from quantitative analysis. Further clinical accuracy studies are necessary to enable quantitative analysis and to confirm the reported positive outcomes of SPG, which showed significantly higher trueness compared to IOS, and higher, although not significant, trueness compared to IOS supported by artificial landmarks.

Considering the 10 eligible in vitro studies, only 7 were included in the quantitative analysis due to the heterogeneity of reported outcomes [20, 22, 23, 24, 27, 28, 29].

All the studies eligible for the quantitative analysis were conducted on one model, except for Pinto et al. who investigated one model but with two implant number configurations (four anterior implants or all six implants), and Sallorenzo et al. who used two models with six implants parallel to each other or with the posterior implants distally angulated. Pinto et al. were only included in the RMS trueness metanalysis, and Sallorenzo et al. in the angular deviation trueness metanalysis. In the RMS precision metanalysis, all the included studies tested the accuracy on one model only [24, 27].

Regarding the effect of the implant number, only Pinto et al. compared the RMS accuracy of the investigated technologies in four versus six implant number configuration, showing that six implants could negatively affect the accuracy compared to four implants (IOS 20.52 μm vs. 40.32 μm; SPG 5.18 μm vs. 13.88 μm). Only this study evaluated the influence of the number variable. Therefore, this outcome can be generalized only with caution.

Sallorenzo et al. were included only in the metanalysis on angular deviation trueness, reporting for the SPG technology a not significant difference between the straight and the angulated implant models (0.280° vs. 0.246°), while IOS performed significantly worse (0.474° vs. 0.841°) on the model with angulated implants. However, only this study evaluated the influence of implant angulation; therefore, this outcome can be generalized only with caution.

The null hypothesis that there is no difference in in vitro trueness angular, and trueness and precision surface deviation between IOS and SPG was rejected, as the effect size was significant (*p* < 0.05) in favor of SPG for all three investigated outcomes.

The meta‐analysis on surface deviation trueness was conducted on four studies, which reported mean IOS and SPG deviation values ranging between 14.8–67.72 μm and 5.18–48.74 μm, respectively, with an effect size of 3.426 in favor of SPG. The meta‐analysis on angular deviation trueness was executed on three studies, reporting mean IOS and SPG deviation values ranging between 0.28°–1.177° and 0.24°–0.81°, respectively, with a significant effect size of 1.199 in favor of SPG. In terms of surface and angular trueness, which is defined as the capability of reproducing faithful digital implant position, SPG performed significantly better compared to IOS.

This significant trueness difference could be attributed to the different technologies of the investigated devices. SPG can record all the implant coordinates and their relationship in the space, simultaneously, thereby avoiding the deviations caused by the stitching process that is inherent in IOS technology. The stitching process is based on consecutive 3D image acquisitions that are linked to each other through a best‐fit algorithm that recognizes common points in the different images. Each image superimposition contributes a small amount of deviation. The greater the number of images to be connected and properly superimposed, the higher the overall deviation. Furthermore, the stitching process can be hindered by the lack or scarcity of stable reference points, a common situation in completely edentulous scenarios.

To overcome this critical issue with IOS, the use of artificial landmarks has been proposed, showing encouraging outcomes in vitro both in terms of accuracy and practicality. However, the application of artificial landmarks for IOS requires an extra step in the procedure and still needs to be validated in vivo [7, 33, 34].

The meta‐analysis on surface deviation precision was conducted on four studies, which reported mean IOS and SPG deviation values ranging between 3.90–37.07 and 0.10–5.46 μm, respectively, with an effect size of 4.892 in favor of SPG. In terms of surface precision, which defines the repeatability of an impression device, SPG performed significantly better compared to IOS, with an effect size even greater than that observed for trueness outcomes.

This extremely significant precision difference can be attributed to the different positioning relative to the patient's mouth, the matter of use, and the different camera technologies characterizing the investigated devices. SPG is an extraoral digital device that requires small movements to focus all the flags screwed onto the implants, making the operator's results less relevant. Moreover, the two infrared stereo cameras of SPG are not affected by factors reported in the literature as potential bias for the performance of the optical cameras in IOS technology (ambient light, saliva, and surface reflection), leading to more repeatable accuracy results.

On the other hand, IOS necessitates the movement of the camera along the arch, so its use is influenced by the scanning strategy and operator skill. Even the same operator could record two digital impressions that greatly differ from each other due to IOS‐related mistakes such as loss of focus or too fast and/or wrong movement of the wand [31, 35]. However, SPG is an extraoral scanning device capable of detecting only the implant coordinates without recording the surrounding soft tissues, dentition, and transmucosal paths. Consequently, another intraoral impression, recorded either with an IOS or through a conventional technique to be later digitized in the lab, is necessary to provide the dental technician with all the anatomical information of the edentulous jaw necessary to prepare a complete master model [33, 35]. A recent systematic review compared SPG and IOS for complete arch implant impressions, including also the conventional technique, and showed comparable accuracy results between the investigated impression techniques [9]. Another recent systematic review also concluded SPG had lower scanning time and higher patient and operator satisfaction when compared with conventional impression and/or IOS [31]. Furthermore, SPG was reported as a reliable alternative to record implant positions, even though one study noted that the SPG system (iCam4D) exhibited precision values ranging from 2 to 203 μm, which is above the clinically recommended threshold [34].

The present systematic review showed that, regardless of the devices and study design, the mean SPG and IOS surface deviations (5.18–48.74 and 14.8–67.72 μm, respectively) and SPG angular deviations (0.24°–0.81°) recorded in vitro were lower than the threshold values of 150 μm and 1°. However, the mean IOS angular deviation values (ranging from 0.28° to 1.177°) were above the threshold of 1°. Generally, a misfit value lower than 150 μm and an angulation deviation of 1° are considered sufficient to avoid prosthetic complications such as screw loosening and fracture [36, 37, 38, 39, 40, 41]. Furthermore, the higher the number of implants to be connected, the lower the amount of misfit accepted, so in the case of complete‐arch implant‐supported prosthesis the misfit tolerance should be considered even lower [42, 43]. Nevertheless, the final misfit of an FDP may be generated not only by the deviations generated during the impression but also by the other steps necessary to fabricate the prosthesis. The manufacturing tolerance is considered another critical factor that could affect the overall fit of the prosthesis and has been considered responsible for the formation of gaps ranging between 20 and 100 μm [44].

The primary limitation of the present study was the paucity of included papers eligible for meta‐analysis; therefore, publication bias tests were not performed. This can be attributed to the relative novelty of the investigated technologies, particularly the SPG devices, and the heterogeneity of the reported outcomes [45, 46].

Furthermore, the evidence presented in this systematic review is predominantly derived from in vitro studies and the results of the meta‐analysis are limited exclusively to these in vitro studies. In vivo trials were not included in the meta‐analysis because of their low number and outcome heterogeneity. Therefore, even though SPG showed higher accuracy, further in vivo comparative studies are necessary to confirm these positive outcomes and validate SPG as the most accurate digital technology for recording complete‐arch implant impressions.

## 5 Conclusion

Within this study's limitations, the following conclusions can be drawn:

1. In the systematic review, SPG demonstrated superior accuracy compared to IOS in 10 of 13 studies. IOS exhibited higher trueness, lower precision, and greater accuracy in two in vitro studies while showing comparable trueness with the assistance of artificial landmarks in one in vivo study.

2. In the meta‐analysis, SPG showed significantly higher angular trueness, surface trueness, and precision compared to IOS, with mean ranges of 0.24°–0.81°, 5.18–48.74 μm, and 0.10–5.46 μm. IOS reported an angular deviation exceeding the 1° threshold required for a passive fit in all three studies.

3. SPG was shown to be a more reliable technology than IOS for complete‐arch digital implant impression. Further clinical trials are required for conclusive evidence. Until then, a rigid prototype try‐in is still recommended.

## Author Contributions

A.P. and L.A. conceived study aims and design; L.A. and P.C. collected the data; A.P., L.A., P.C., and A.L. analyzed the data; A.P., L.A., P.C., J.L., and H.‐L.W. led the writing; A.P., J.L., and H.‐L.W. approved the submission.

## Conflicts of Interest

The authors declare no conflicts of interest.

## Data Availability

The data that support the findings of this study are available from the corresponding author upon reasonable request.
